# Round pneumonia: a rare condition mimicking bronchogenic carcinoma. Case report and review of the literature

**DOI:** 10.1590/S1516-31802008000400010

**Published:** 2008-07-03

**Authors:** José de Jesus Peixoto Camargo, Spencer Marcantonio Camargo, Tiago Noguchi Machuca, Fabíola Adélia Perin

**Keywords:** Pneumonia, Lung neoplasm, Lung diseases, Medical imaging, Lung, Pneumonia, Neoplasias pulmonares, Pneumopatias, Diagnóstico por imagem, Pulmão

## Abstract

**CONTEXT:**

Round pneumonia is a condition usually described in children, with few reports addressing adult patients. It is an oval-shaped consolidation that, due to its radiological appearance, simulates bronchogenic carcinoma. Its evolution tends to be benign, although diagnostic dilemmas have sometimes required exploratory thoracotomy. Deaths caused by this condition have even been reported. To the best of our knowledge, there have been 31 previous cases of round pneumonia in adults reported in the English and Portuguese-language literature, of which only one was completely asymptomatic.

**CASE REPORT:**

The case of a 54-year-old female patient presenting a lung mass found on routine imaging evaluation is reported. Respiratory symptoms and signs were absent, but the patient had a significant history of smoking. Her physical examination gave normal results. On chest radiographs, a mass located in the middle third of the right lung was observed. Three weeks after the initial evaluation, the patient was admitted for a complete evaluation and for staging of a pulmonary malignancy, but repeated chest radiographs showed complete resolution.

## RESUMO

**CONTEXTO:**

A pneumonia redonda é uma condição encontrada normalmente em crianças, sendo que relatos envolvendo adultos são raros. Ela é definida como uma consolidação pulmonar ovalada que, pelo aspecto radiográfico, simula o carcinoma broncogênico. Sua evolução tende a ser benigna, entretanto, dificuldades diagnósticas demandando toracotomia exploradora e até mesmo óbitos têm sido descritos. Em revisão de literatura de língua inglesa e portuguesa, foram encontrados somente 31 casos relatados, com apenas um único caso de paciente com apresentação assintomática.

**RELATO DE CASO:**

Os autores relatam o caso de uma paciente de 54 anos com achado de massa pulmonar em exame radiológico de rotina. A paciente negava sinais e sintomas respiratórios, entretanto referia uma importante história de tabagismo. No exame físico, nada digno de nota. Na radiografia de tórax se observava uma lesão arredondada no terço médio do pulmão direito. Três semanas após a avaliação inicial, a paciente foi internada para completar a avaliação e estadiamento de uma possível neoplasia pulmonar, entretanto, o raio-x da entrada mostrou completa resolução do processo.

## INTRODUCTION

In 1964, Greenfield and Gyepes were the first to draw attention to a pneumonia variant that simulated bronchogenic carcinoma. In their report, they stated that it required extensive diagnostic workup, including thoracotomy in one case.^1^ In 1973, Rose and Ward reviewed their experience with 21 similar cases affecting children and stressed the importance of recognizing this clinical entity known as round pneumonia (RP).^2^

Subsequent studies reinforced the notion that RP is a condition that occurs in children, and pediatricians became more familiar with dealing with it.^3^ Reports involving adults remain rare. In a search in the Medical Literature Analysis and Retrieval System Online (Medline) database restricted to the English and Portuguese-language literature, we identified 31 cases, of which only one patient presented without complaints.^1,4-15^ (Table 1). The present study describes the second case of asymptomatic RP and reviews the pertinent literature.

**Table 1:** Reports on round pneumonia among adults in the literature

Study	n	Age*	Location	Chief complaints	Outcome
Greenfield and Gyepes1	8	38.7 years (31-68)	URL – 3 MRL – 2 LRL – 1 LLL – 2	dyspnea, fever	resolved: 7 death: 1
Sproul4	1	26 years	ULL	coughing, chills	resolved
Soubani and Epstein5	2	36-37 years	ULL – 1 URL – 1	fever, coughing dyspnea, hemoptysis	death: 1 resolved: 1
Pandya et al.6	1	22 years	MRL – LRL	coughing, fever	resolved
Millard et al.7	1	57 years	LRL	fever, coughing, dyspnea	resolved
Hershey and Panaro8	3	33.6 years (21-58)	URL LLL ULL	fever, dyspnea cough asymptomatic	resolved resolved resolved
Lossos and Breuer9	1	37 years	ULL	chest pain	resolved
Durning et al.10	1	58 years	LLL	coughing, dyspnea, fever	resolved
Zinkernagel et al.11	1	33 years	URL	generalized malaise	resolved
Jardim et al.12	1	57 years	LRL	coughing, fever	resolved
Wan et al.13	8	32.5 years (18-47)	R Lung – 7 L Lung – 1	fever, coughing, dyspnea	resolved: 7 death: 1
Zylberman et al.14	2	24-34 years	URL – 2	fever, coughing, dyspnea	resolved 2
Shie et al.15	1	75 years	ULL	fever, hemoptysis	resolved
Present case	1	54 years	LRL	asymptomatic	resolved

*URL = upper right lobe; MRL = middle right lobe; LRL = lower right lobe; LLL = lower left lobe; ULL = upper left lobe; R = right; L = left.*

* *In studies with more than two cases, age is reported as the mean, with the range in brackets.*

## CASE REPORT

**Figure 1:**
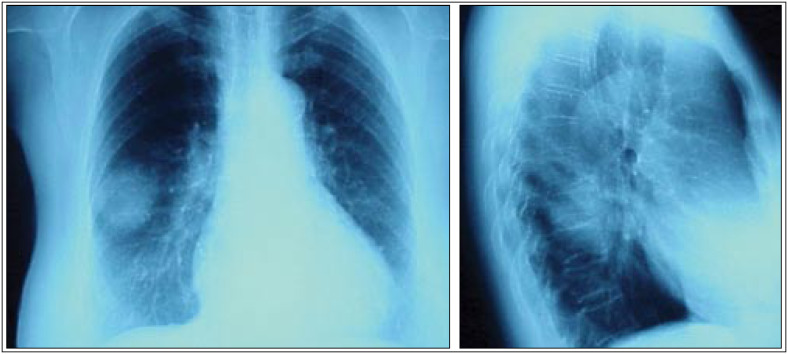
Posteroanterior and lateral chest radiograph views, showing a round mass located in the upper segment of the lower right lobe.

**Figure 2:**
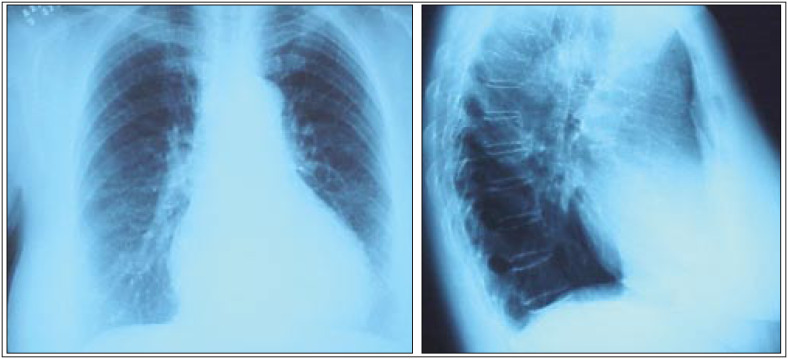
Posteroanterior and lateral chest radiographs produced on admission three weeks after the initial films. Complete resolution is clearly observed.

A 57-year-old female patient was referred to us with a pulmonary mass found on a routine chest radiograph. She had no complaints and her medical history had been uneventful, but she had a 30 pack-year history of smoking. On physical examination, there was no cervical adenopathy. Thorax percussion gave normal results on auscultation. A chest radiograph displayed a right lower-lobe mass, located in the upper segment on lateral view (Figure 1). Three weeks later, the patient was admitted to hospital for a complete evaluation and for staging to be performed, but a new chest radiograph revealed that the process had resolved (Figure 2).

## DISCUSSION

RP has been defined as an oval or round-shaped consolidation distributed in a nonsegmental pattern. Its pathogenesis is unknown, although it is often hypothesized that atypical dissemination of the exudative fluid of early pneumonia through interalveolar communication (the so-called pores of Kohn and channels of Lambert) is responsible for the nonsegmental pattern and centrifugal distribution that sharply distinguish such cases from healthy lung tissue.^5^

Pediatric studies have characterized the clinical features of RP. Patients usually present with acute febrile illness and the chief complaints include coughing, dyspnea and chest pain. Physical signs of parenchymal consolidation such as dullness to percussion and rales on auscultation are frequently present. The most striking laboratory finding is leukocytosis, and there is generally a clinical-radiological response to a course of antibiotics.^2,16^

According to Zinkernagel et al., the higher incidence of RP among children is mainly due to the closely apposed connective tissue septa and smaller alveoli in this population, which contributes towards the formation of more compact and confluent consolidations.^11^ Furthermore, disease progression tends to be slower, thus favoring radiological detection of the oval-shaped images.

Most cases of RP are attributed to *Streptococcus pneumoniae*.^11^ However, several other agents have been described, including typical agents such as *Klebsiella pneumoniae,*^5^ *Haemophilus influenzae*^4^ and *Mycobacterium tuberculosis*;^11^ atypical agents such as *Chlamydia psitacci*^14^ and *Coxiella burnetti* (*Rickettsiae*);^17^ and viral agents such as coronavirus.^13^

The literature regarding RP in adults is summarized in Table 1. Topographically, as shown by our case, the right lung is more frequently involved, accounting for 22 of the 31 reported cases. No predilection for the upper or lower lobe is observed.^1,4-15^

It is believed that the true incidence of RP is much higher than reported, and that most cases are adequately treated without ever undergoing radiological examination.*18* This picture is well illustrated by the report by Wan et al., while evaluating an outbreak of severe acute respiratory syndrome (SARS) in Taiwan.^13^ Because of the high virulence and infectivity of coronavirus, radiographic follow-up was extensively performed. This yielded a surprisingly high incidence of RP: in 29% of their 28 SARS cases.

RP often follows a benign course, with resolution after a course of antibiotics, or even spontaneously, as in our case. Nevertheless, fatalities have been described. In the report by Greenfield and Gyepes, a 68-year-old man presenting dyspnea, coughing and fever died despite antibiotic treatment.^1^ In the study by Soubani and Epstein, two young men (one with alcoholic liver disease and the other with a history of intravenous drug abuse) presented respiratory failure and required mechanical ventilatory support. The one with liver disease developed a fulminant course and died within 24 hours of admission.^5^ Lastly, in the SARS report cited above, two patients also underwent endotracheal intubation because of respiratory failure and one of them died after nine days of hospitalization.^13^ As would be expected, mortality in RP cases is related to well-known conditions such as advanced age, immunosuppression and extremely virulent agents.

The importance of recognizing RP in clinical practice lies in its radiological appearance, which often mimics lung cancer. Complaints from patients usually point towards an infectious disease but, not only in our case but also in one of Hershey and Panaro's, the patients were asymptomatic.^8^ It should be noted that, unlike in our case, RP usually affects patients at a younger age. The mean age in the cited studies was 40.9 years and this information may help to raise the suspicion that this is an inflammatory rather than a neoplastic process. However, only radiological resolution can safely rule out the hypothesis of neoplasia. Even imaging techniques of a more advanced nature have failed to differentiate RP from lung cancer, such that similar patterns of F18-FDG uptake have been described from positron emission tomography/computed tomography scans.^15^

In summary, RP is a known condition affecting children but reports among adults are rare. It usually follows a benign course, but deaths have been reported among high-risk groups. This study describes an atypical case of RP. Furthermore, diagnostic pitfalls are discussed and the concept that only complete radiological resolution can safely rule out lung cancer is highlighted.

## CONCLUSION

Even though RP usually affects young patients and presents with the signs and symptoms of an infectious disease, older patients with risk factors for lung cancer and no clinical evidence of infection can also present this condition.
